# Acute memory and psychotomimetic effects of cannabis and tobacco both ‘joint’ and individually: a placebo-controlled trial

**DOI:** 10.1017/S0033291717001222

**Published:** 2017-05-31

**Authors:** C. Hindocha, T. P. Freeman, J. X. Xia, N. D. C. Shaban, H. V. Curran

**Affiliations:** 1 Clincial Psychopharmacology Unit, University College London, Gower St, London, UK; 2 The Sophie Davis School of Biomedical Education, The City College of New York, NY, USA

**Keywords:** Cannabis, co-administration, drug-interaction, marijuana, memory, psychosis, tobacco

## Abstract

**Background:**

Cannabis and tobacco have contrasting cognitive effects. Smoking cannabis with tobacco is prevalent in many countries and although this may well influence cognitive and mental health outcomes, the possibility has rarely been investigated in human experimental psychopharmacological research.

**Method:**

The individual and interactive effects of cannabis and tobacco were evaluated in 24 non-dependent cannabis and tobacco smokers in a randomized, placebo-controlled, double-blind, 2 (cannabis, placebo) × 2 (tobacco, placebo) crossover design. Verbal memory (prose recall), working memory (WM) performance including maintenance, manipulation and attention (N-back), psychotomimetic, subjective and cardiovascular measures were recorded on each of four sessions.

**Results:**

Cannabis alone impaired verbal memory. *A priori* contrasts indicated that tobacco offset the effects of cannabis on delayed recall. However, this was not supported by linear mixed model analysis. Cannabis load-dependently impaired WM. By contrast, tobacco improved WM across all load levels. The acute psychotomimetic effects and ratings of ‘stoned’ and ‘dizzy’ induced by cannabis were not altered by tobacco. Cannabis and tobacco had independent effects on increasing heart rate and interacting effects on increasing diastolic blood pressure.

**Conclusions:**

Relative to placebo, acute cannabis impaired verbal memory and WM. Tobacco enhanced performance on WM, independently of cannabis. Moreover, we found some preliminary evidence that tobacco may offset the effects of cannabis on delayed, but not immediate, verbal recall. In contrast, the psychotomimetic and subjective effects of cannabis were unaffected by tobacco co-administration. By reducing the cognitive impairment from cannabis, tobacco co-administration may perpetuate use despite adverse health consequences.

## Introduction

Cannabis and tobacco, two of the world's most commonly used drugs, are frequently co-administered together in ‘joints’ or ‘spliffs’ (Hindocha *et al.* 2016). Delta-9-tetrahydrocannabinol (THC) and nicotine, respectively are these drugs primary psychoactive components. Cannabinoid receptors (CB1R) and nicotine acetylcholine receptors (nAChRs) are both densely populated in the hippocampus and the amygdala, suggesting a potential neurobiological overlap (Herkenham *et al.* 1990; Picciotto *et al.* 2000) for the effects of these two drugs on memory. This is evident behaviourally as THC and nicotine have opposite effects on memory and cognition in humans with THC impairing and nicotine facilitating performance (Gray *et al.* 1996; Levin & Simon, 1998; Curran *et al.* 2002; D'Souza *et al.* 2004; Levin *et al.* 2006; Morrison *et al.* 2009; Heishman *et al.* 2010; Bossong *et al.* 2012). Preclinical data suggest a relationship between the endocannabinoid system and the cholinergic system. Full or partial agonists of the CB1R, including THC, increase nicotine conditioned place preference (Valjent *et al.* 2002) and self-administration (Gamaleddin *et al.* 2012) whereas antagonists at this receptor (e.g. rimonabant) decrease these behaviours (Le Foll & Goldberg, 2004; Cohen *et al.* 2005; Forget *et al.* 2005; Shoaib, 2008). Further nicotine and THC both interact with mesolimbic dopaminergic pathways potentially modulating reward-related processes in addiction (Rowell *et al.* 1987; Fernandez-Ruiz *et al.* 2010).

Acutely, cannabis produces a profile of cognitive impairment, similar to that associated with schizophrenia, and particularly in the realms of working and episodic memory (Fletcher & Honey, 2006; Broyd *et al.* 2016; Curran *et al.* 2016). Deficits in episodic memory are some of the most robust findings reported (Crane *et al.* 2013). Dose-dependent (Hart *et al.* 2010) effects of THC on working memory are also consistently reported (Hunault *et al.* 2009; Ramaekers *et al.* 2009) and are specifically related to the manipulation rather than the maintenance of information. Nicotine, in contrast, improves memory in both smokers and non-smokers (Heishman *et al.* 2010). It has been hypothesised that tobacco might compensate for some of the negative effects of cannabis (Rabin & George, 2015). In support of this, individuals smoking cannabis and cigarettes have less episodic memory impairment when drug free compared with cannabis users alone (Schuster *et al.* 2015), but experience worse cognitive withdrawal symptoms from tobacco in regards to episodic and working memory (Jacobsen *et al.* 2007). Moreover, an ecological momentary assessment study found that when cannabis and tobacco are combined, working memory performance was better in comparison with cannabis alone (Schuster *et al.* 2016). However, to our knowledge, no controlled studies have examined whether tobacco can offset the cognitive impairing effects of cannabis.

Importantly, epidemiological research has implicated both cannabis and tobacco as independent risk factors for psychosis (Moore *et al.* 2007; Gurillo *et al.* 2015). It is clear that both cigarette smoking and problematic cannabis use are highly prevalent in people with schizophrenia in epidemiological research (De Leon & Diaz, 2005; Koskinen *et al.* 2010). However, it can be extremely challenging to dissociate the role of cannabis from tobacco in these studies due to the high co-occurrence of their use both i.e. cannabis users are more likely to smoke cigarettes, and cannabis and tobacco are often combined into joints and smoked together (Gage *et al.* 2014). Acutely, cannabis/THC induces psychotic-like effects, including paranoia, disorganised thinking and hallucinations. However, there is no such experimental evidence that nicotine/tobacco induces or exacerbates psychotic- symptoms acutely. One study investigated the acute effect of a nicotine patch on cannabis induced psychotomimetic effects (using the Addiction Research Center Inventory: LSD subscale) where nicotine had no effect on THC (Penetar *et al.* 2005). However, this study lacks the ecological administration method of ‘joints’ and did not use a scale specific to the psychotomimetic drug effects (Mason *et al.* 2008). Thus, given the high prevalence of use of cannabis and tobacco, it is necessary to understand the interactive effects on psychotic-like symptoms induced by cannabis.

This study, therefore, aimed to investigate the individual and combined effects of cannabis and tobacco on episodic and working memory, and psychotic-like experiences. We hypothesised that tobacco would acutely counteract the negative effects of cannabis on working and episodic memory and will directly test with an *a priori comparison of* the combination of cannabis + tobacco and cannabis alone. We also hypothesised that cannabis would increase psychotic-like symptoms; how nicotine would influence these was exploratory given the dearth of previous relevant research.

## Methods and materials

### Design and participants

A randomised double-blind, placebo-controlled, four-way, crossover trial was used to evaluate the acute effects of cannabis and tobacco, both alone and combined (Table 1). Participants attended four sessions, separated by at least 1-week washout (as this is  ⩾ 3 times elimination half-life of THC) (D'Souza *et al.* 2008; Hindocha *et al.* 2015). Washout of nicotine was confirmed by Carbon Monoxide (CO) ⩽ 6 (Bedfont Micro Smokerlyser, Bedfont Scientific Ltd, Bedfont, UK). Order of treatment was determined by a balanced Latin square. All participants provided written, informed consent on each occasion. Ethical approval was given by the UCL Ethics Committee.

**Table 1.** Cannabis and tobacco doses in the study drug and their matched placebos (see Fig. 1)

Drug	Condition	Description
Cannabis	Active	66.67 mg Bedrobinol (16.1% THC and <1% CBD)
	Matched placebo	66.67 mg Placebo (derived from Bedrocan; 0.07% THC)
Tobacco	Active	311 mg Marlboro Red (15.48 mg nicotine, 16 mg tar, 0.8 mg nicotine yield)
	Matched placebo	311 mg denicotinized tobacco (Magic 0, 0.04 mg/g nicotine)

Medically and psychiatrically healthy, non-dependent but experienced, cannabis and tobacco users were recruited. A flowchart of participant recruitment can be found in the online Supplementary Materials (see online Supplementary Fig. S2).

### Power calculation

Power was informed by a previous four-way crossover trial examining interactive effects of THC and Cannabidiol (CBD) (*d* = 0.5; based on a *t* test of THC + CBD attenuating negative effects of THC (Hindocha *et al.* 2015)). This estimated a sample size of 24 participants with complete data would achieve power of *d* = 0.5 to detect such effects with an alpha of 0.05 (G*power version 3.1.9.2) (Faul *et al.* 2007). This was also appropriate for completely balancing the order of the four treatments completed the study as 24 = 4 factorial.

### Inclusion criteria

Inclusion criteria were: (i) age 18–60 years, (ii) regular (⩾ once per month and  ⩽ 3 times a week) use of cannabis and tobacco in joints for the last 6 months, (iii) self-reported (SR) ability to smoke one whole ‘standard’ joint, (iv) normal or corrected-to-normal vision, (v) fluent English, (vi) SR abstinence from tobacco, cannabis, alcohol and other drugs for ⩾ 12 h prior to each session, (vii) alveolar CO ⩽ 6 ppm to confirm no recent smoking on each test day (Cooper & Haney, 2009). Exclusion criteria were (i) scoring ⩾ 3 on the cannabis Severity of Dependence Scale (SDS; Gossop *et al.* 1995), (ii) treatment-seeking for cannabis, tobacco use, or currently using nicotine replacement therapy or other cessation pharmacotherapy; (iii) smoking ⩾ 10 cigarettes a day or scoring ⩾ 4 on the Fagerstrom Test of Nicotine Dependence (FTND; Heatherton *et al.* 1991) consistent with previous research (Agrawal *et al.* 2009), (iv) first cigarette smoked within the first 3 h after waking (to ensure cognitive results were not simply due to reversal of withdrawal from tobacco (Jarvik *et al.* 2000)), (v) significant respiratory, physical or clinically diagnosed learning disorders, (vi) SR diagnosis of a psychotic disorder (or a first degree family member with a psychotic disorder), or substance use disorder, or (vii) SR use of illicit substance use other than cannabis more than once per week.

### Drug administration (Fig. 1/Table 1)

We compared the effects of (a) active cannabis + active tobacco (CAN-TOB) (b) active cannabis + placebo tobacco (CAN), (c) placebo cannabis + active tobacco (TOB), (d) placebo cannabis + placebo tobacco (no active drug) (PLACEBO). The dose of cannabis specified in Table 1 was based on previous experimental studies reporting robust subjective, cardiovascular, psychotomimetic and memory impairing effects (Lawn *et al.* 2016; Mokrysz *et al.* 2016). This dose of tobacco reliably produces peak plasma nicotine levels >20 ng/ml (Mendelson *et al.* 2003, 2005) and is similar to a standard cannabis + tobacco joint (Hunault *et al.* 2009; Van Der Pol *et al.* 2014). Placebo tobacco was the same dose of Very Low Nicotine (VLN; typically referred to as denicotinized) tobacco (Magic 0; XXII Century Group Ltd).

**Fig. 1.**
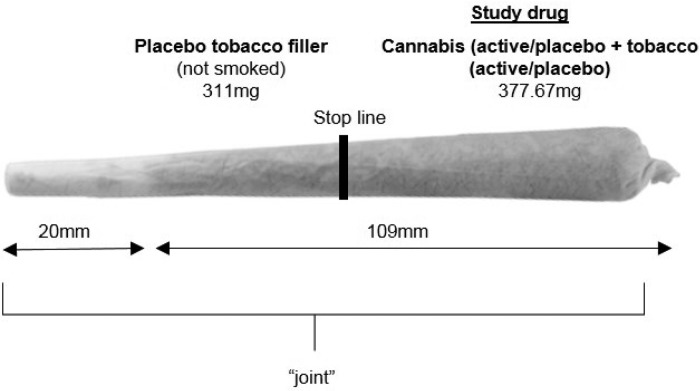
Drug administration was conducted using ‘joints’, the most common method of administering cannabis (Hindocha *et al.* 2016). ‘Study drug’ region contained a mixture of 66.67 mg cannabis (active or placebo) and 311 mg tobacco (active or placebo) dependent on condition (see Table 1). The ‘placebo tobacco filler’ region contained 311 mg of placebo tobacco at the bottom of the joint (nearest to the mouth), which was not smoked. This filler was added to improve compliance with the fixed inhalation procedure (see online Supplementary Materials), as puff volume typically decreases towards the end of the joint, probably due to rising heat (Van Der Pol *et al.* 2014). The stop line is the point at which participants stopped smoking the joint, separating the two regions. It was marked 1 cm after the ‘study drug’ to ensure complete inhalation.

### Procedure

After telephone screening, eligible participants attended a baseline session involving further screening and task training and then four experimental sessions. Each experimental session began with pre-drug Visual Analogue Scales (VAS), physiological measures and a CO measurement to check abstinence from smoking. Participants then listened to a passage of prose and were required to immediately recall its content (story 1). Drug administration took place immediately after this (see online Supplementary Materials for full details on smoking procedure). Thirty-five minutes after drug administration, participants listened to a second passage of prose and immediately recalled its contents (story 2). Delayed recall of story 1 and 2 occurred approximately 55 min after drug administration. Participants completed the N-back and Psychotomimetic States Inventory (PSI; Mason *et al.* 2008) at 21 and 45 min, respectively (see the assessment flowchart online Supplementary Fig. S2). Other tasks that are not reported here took place in the intervening time. Participants were reimbursed £60 for their time and debriefed fully.

### Assessments

#### Baseline measures

Participants completed the Beck Depression Inventory (BDI; Beck *et al.* 1996), Spielberger State-Trait Anxiety Inventory (STAI; Spielberger *et al.* 1970), Schizotypal Personality Questionnaire (SPQ; Raine, 1991) and a detailed drug history including questions about cannabis and tobacco co-use. CO, heart rate (HR), systolic and diastolic blood pressure (BP) and subjective effects were measured pre- (−10) and at 10, 30, 40 and 70 min’ post-drug.

#### Cognitive measures

The Prose Recall subtest of the Rivermead Behavioral Memory Test (Wilson *et al.* 1991) taps episodic memory. Participants were required to listen to a passage of prose (a 30 s news bulletin) and recall its contents both immediately and after a delay. The first story (1) was heard before drug administration, followed by immediate recall. The second story (2) was heard 35 min after drug administration. Delayed recall of both was approximately 55 min after drug administration. This design was chosen to dissociate drug effects on encoding from retrieval (Fletcher & Honey, 2006). Drug effects on encoding would be evidenced by story 2 (both immediate and delayed) being affected, but not story 1 (i.e. a drug × story interaction). If there were drug effects on retrieval, this would be evidenced by a difference on delayed, but not immediate, recall of story 1 (i.e. a drug × story × delay interaction). Each story contained 21 ‘idea units’ and scoring was systematic. The primary outcome is the mean number of idea units recalled. The eight versions were counterbalanced across drug and design.

A Spatial N-back was used to assess spatial working memory. Visual stimuli (smiley faces) appeared in one of six different locations around a central fixation cross on the computer screen, in a sequential order (Freeman *et al.* 2012; Morgan *et al.* 2014). Participants responded by pressing a ‘Yes’ or ‘No’ key according to whether (a) the stimuli appeared in a pre-defined location (zero back; attentional control), (b) whether the stimulus was in the same position as the stimulus one before (1-back), and subsequently, (c) two before (2-back). Four versions of the task were counterbalanced across drug and design and reaction time and accuracy were recorded.

### Psychotomimetic effects

The Psychotomimetic States Inventory (PSI) (Mason *et al.* 2008) was used to assess current schizotypal symptoms. It has 48 items and is specifically designed to measure drug-induced changes in psychotic-like symptoms. It has previously been shown to be sensitive to cannabis-induced psychotomimetic effects and has better test-retest reliability than the Clinician Administered Dissociative States Scale (CADSS; De Simoni *et al.* 2013).

### Statistical analysis

Data were analysed using IBM Statistical Package for Social Sciences (IBM SPSS version 23). Outliers more than 2.5 standard deviations (s.d.) from the sample mean were replaced with a score falling within 2.5 s.d. Normality was explored using visual inspection of diagnostic plots. Data for the Prose Recall, N-back and PSI was analysed using linear mixed models, which included a random intercept for subjects and two within subjects factors of drug: Cannabis (placebo; active) and Tobacco (placebo; active). Additional task-specific factors of Story (1, 2) and Delay (immediate, delayed) for the prose recall and Load (0, 1, 2) for N-back outcomes (correct responses, RT, *d*′, C). VAS scores and physiological factors (HR, BP, CO) had an additional task-specific factor of Time (1 (predrug) *v.* 2, 3, 4, 5 (postdrug)). The unstructured variance-covariance structure was selected following D'Souza *et al.* (2012). Interactions were explored via Bonferroni corrected *post-hoc* comparisons locally within hypotheses but not across hypotheses (D'Souza *et al.* 2012). All descriptive statistics for linear mixed models are estimated marginal means and standard error. *d*′ and C (N-back) were calculated using signal detection analysis (Snodgrass & Corwin, 1988). The loglinear approach was used to account for perfect scores (Hautus, 1995; Stanislaw & Todorov, 1999). Maintenance was calculated as 1-back minus 0-back and manipulation as 2-back minus 1 back.

## Results

### Demographics and drug history

A total of 24 participants (12 women), with a mean ± s.d. age of 24.46 ± 3.96 completed the study. They had minimal dependence on cannabis (SDS: 0.67 ± 0.92 (range: 0–3)) and tobacco (FTND: 0.33 ± 0.64 (range: 0–2)). Those who smoked daily (*N* = 6) reported smoking their first cigarette 5.91 ± 3.01 h after waking. Baseline questionnaire scores were: STAI trait 35.75 ± 8.60; BDI 6.17 ± 5.82; SPQ 19.14 ± 10.83. Other drug use apart from alcohol was minimal (see online Supplementary Table S1).

### Assessments

There were no significant pre-drug differences between the four drug conditions in VAS scores, HR, BP, CO or Short State Anxiety Inventory (SSAI; Marteau & Bekker, 1992). There were no significant differences on time taken or a number of puffs between drug conditions (online Supplementary Table S2).

### Prose recall (Fig. 2)

There was a cannabis × story interaction (*F*_1,23_ = 18.51, *p* < 0.001) and a story × delay interaction (*F*_1,23_ = 26.60, *p* < 0.001). There were also main effects of cannabis (*F*_1,23_ = 10.65, *p* = 0.003) and delay (*F*_1,23_ = 107.58, *p* < 0.001) but not of tobacco or story. No significant interaction between cannabis and tobacco emerged (*F*_1,23_ = 0.812, *p* = 0.317).

**Fig. 2.**
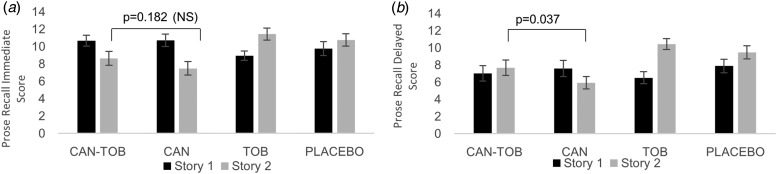
(*a* and *b*) Immediate recall (*a*) and delayed recall (*b*) under each drug condition for both story 1 (where encoding was not intoxicated) and story 2 (where encoding was intoxicated). Under delayed recall, for story 2, we found CAN-TOB in comparison with CAN, improves delayed recall but this was not the case for immediate recall, therefore suggesting effects on retrieval of information that had previously been successfully encoded. Error bars show ±s.e.m.

The cannabis × story interaction showed poorer recall following cannabis (M: 7.71, s.e.: 0.63) for story 2 in comparison with placebo (M: 10.44, s.e.: 0.68) (*p <* 0.001) but not for story 1 (*p* = 0.324). Under placebo cannabis, there was greater recall for story 2 (M: 10.44, s.e.: 0.68) in comparison with story 1 (M: 8.45, s.e.: 0.51) (*p <* 0.001). By contrast, for active cannabis, there was greater recall on story 1 (M: 8.94, s.e.: 0.62), in comparison with story 2 (M: 7.71, s.e.: 0.63) (*p* = 0.019).

To test our *a priori* hypothesis that tobacco compensates for the detrimental effect of cannabis on memory we compared the difference between CAN-TOB on immediate and delayed recall for story 2 with critical *t* tests (Fig. 2*a*). On immediate recall, there was no difference (*t*_23_ = 1.38, *p* = 0.182) but on delayed recall, scores were significantly higher after CAN-TOB compared with CAN; the mean difference was 1.75 idea units (s.d.: 3.87) (*t*_23_ = 2.21, *p* = 0.037, *d* = 0.5) (Fig. 2*b*).

The story × delay interaction showed that story 2 (M: 8.47, s.e.: 0.61) was remembered better than story 1 (M: 7.31, s.e.: 0.61) after the delay (*p* = 0.007) but there was no difference for immediate recall (*p* = 0.360), which suggests a recency effect. The main effect of cannabis (M: 8.32, s.e.: 0.56) clearly showed that cannabis impaired recall in comparison with placebo (M: 9.45, s.e.: 0.54). The main effect of delay simply showed delayed recall (M: 7.89, s.e.: 0.58) was poorer than immediate recall (M: 9.88, s.e.: 0.48).

### N-back

#### Correct responses (Fig. 3*a*, *b*)

**Fig. 3.**
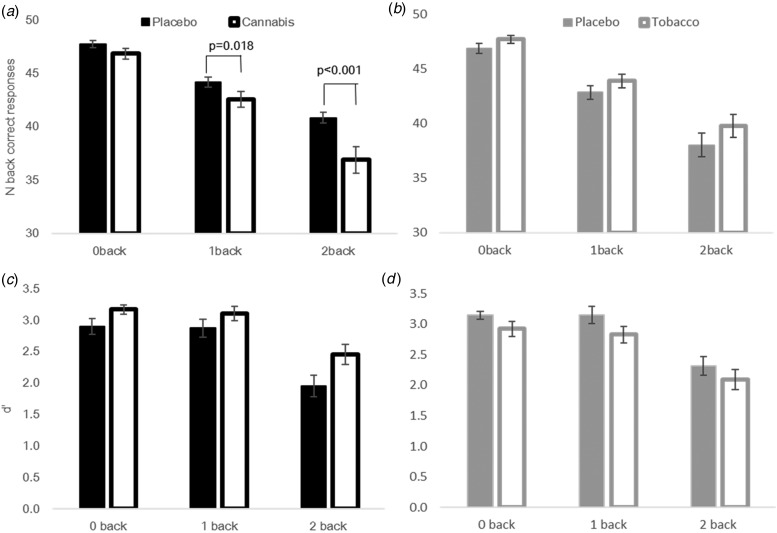
(*a–d*) Number of correct responses (*a* & *b*) and *d*′ (*c* & *d*) for cannabis *v.* placebo (*a* & *c*) and tobacco *v.* placebo (*b* & *d*) for the N-back. Error bars show ±s.e.m.

There was a cannabis × load interaction (*F*_2,23_ = 4.82, *p* = 0.018), which showed that cannabis impaired the 1- and 2-back but not the zero-back (Fig. 3*a*; also online Supplementary Table S3). A main effect of cannabis (*F*_1,23_ = 15.93, *p* = 0.001), reflected better performance on placebo than cannabis and a main effect of tobacco (*F*_1,23_ = 4.88, *p* = 0.037) reflected better performance on active tobacco (M: 43.77, s.e.: 0.55) than placebo (M: 42.58, s.e.: 0.56) across all load conditions (Fig. 3*b*). A main effect of load (*F*_2,23_ = 43.42, *p <* 0.001) reflected better performance on 0-back than 1- and 2-back, respectively. No significant interaction between cannabis and tobacco emerged. The critical a *priori t* test between CAN-TOB and CAN on N-back correct responses across all loads was not significant (*p* > 0.5).

#### Signal detection analysis (Fig. 3*c*, *d*)

*d*′: there was a main effect of cannabis (*F*_1,23_ = 14.48, *p* < 0.001) where it reduced discriminability in comparison with placebo (Fig. 3*c*), a main effect of tobacco (*F*_1,23_ = 8.25, *p* = 0.009) where tobacco increased discriminability in comparison with placebo (Fig. 3*d*) and a main effect of load, (*F*_2,23_ = 28.33, *p* < 0.001). The highest discriminability was for the 0-back, followed by the 1-back, followed by the 2-back and there were no significant interactions. The critical apriori *t* test between CAN-TOB and CAN on *d*′ averaging over all loads showed a trend towards higher scores with CAN-TOB in comparison with CAN (*t*_23_ = 2.00, *p* = 0.059, *d* = 0.47).

*Criterion* (*C*): there was a main effect of load (*F*_2,23_ = 245.90, *p* < 0.001) whereby the criterion was higher for the 0-back (M: 0.50, s.e.: 0.02), followed by the 1-back (M: −0.04, s.e.: 0.02) and 2-back (M: −0.06, s.e.: 0.03).

#### Reaction time

There was a cannabis × load interaction (*F*_2,23_ = 8.82, *p* < 0.001), which showed that cannabis impaired the 2-back in comparison with placebo (*p* = 0.005) but not the 1-back (*p* = 0.214) or the 0 back (*p* = 0.979). There was a main effect of load (*F*_2,23_ = 68.90, *p <* 0.001), which showed increasing RT across load. There were no main effects or interactions with tobacco.

#### Manipulation and maintenance

A main effect of cannabis on manipulation (*F*_1,23_ = 5.86, *p* = 0.024) showed cannabis impaired manipulation (M: −5.67, s.e.: 1.04) in comparison with placebo (M: −3.27, s.e.: 0.77); there were no other effects or interactions. No main effects or interactions emerged for maintenance.

### Psychotomimetic states inventory

A main effect of cannabis (*F*_1,33_ = 33.01, *p* < 0.001) showed cannabis (M: 32.04, s.e.: 3.53) markedly increased PSI scores in comparison with placebo (M: 13.85, s.e.: 1.76); there were no other effects or interactions. The same pattern of results emerged when including PSI subscale as an additional factor. Schizotypy has previously been found to predict acute psychotomimetic response to cannabis; therefore, we added SPQ score as an additional covariate. This did not reveal any interactions between SPQ score and drug effect on the PSI.

### Physiological measures

#### Carbon monoxide (Fig. 4*a*)

There was a main effect of cannabis (*F*_1,161_ = 4.32, *p* = 0.039), which showed that under active cannabis, participants had a lower CO than under placebo cannabis. There was also a main effect of time (*F*_1,161_ = 415.49, *p* < 0.001).

**Fig. 4.**
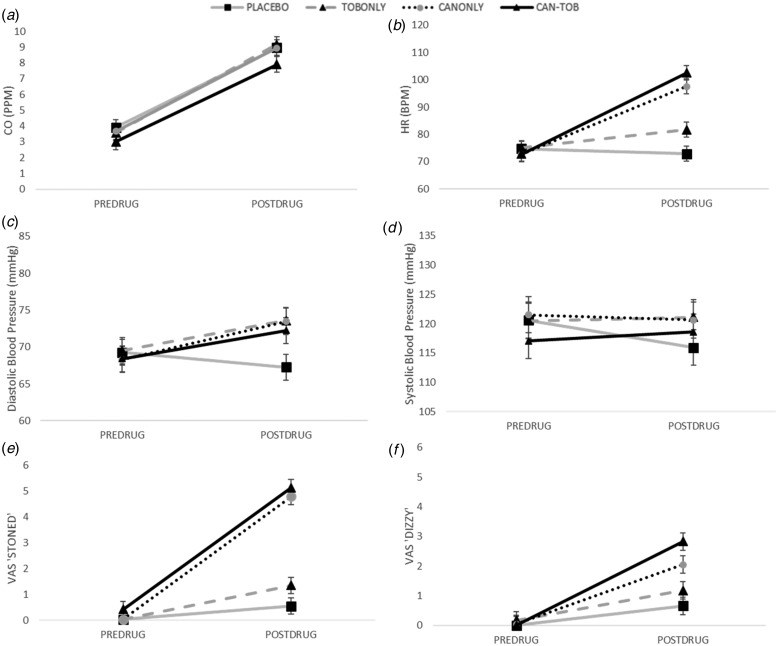
(*a–f*) carbon monoxide (CO), cardiovascular (heart rate (HR), systolic and diastolic blood pressure (mmHg)) and self-reported effects for stoned and dizzy for all time points before (T1) and after (T2–T5) each drug administration. Error bars show ±s.e.m.

#### Heart rate (Fig. 4*b*)

A cannabis × time interaction (*F*_1,161_ = 62.88, *p* < 0.001) revealed a significant increase on active cannabis, compared with placebo cannabis, post-drug administration (MDiff: 22.71, s.e.: 2.22, *p <* 0.001), but no difference pre-drug. It also revealed an increase between pre-and post-drug for active cannabis (MDiff: 27.31, s.e.: 2.20, *p <* 0.001), but not for placebo cannabis. A tobacco × time interaction (*F*_1,161_ = 4.49, *p* = 0.036) revealed a significant increase between tobacco and placebo, post-drug (MDiff: 6.88, s.e.: 2.20; *p* = 0.002). There was no difference between tobacco and placebo pre-drug. Under both placebo and active tobacco, there was an increase in HR from pre- to post-drug (placebo tobacco MDiff: 11.53, s.e.: 2.20; *p <* 0.001, active tobacco MDiff: 18.18, s.e.: 2.20, *p <* 0.001). There were main effects of cannabis (*F*_1,161_ = 42.73, *p* < 0.001), tobacco (*F*_1,161_ = 5.125, *p* = 0.025) and time (*F*_1,161_ = 89.53, *p* < 0.001).

#### Blood pressure (Fig. 4*c*, *d*)

For diastolic blood pressure, there was a cannabis × tobacco × time interaction (*F*_1,161_ = 5.56, *p* = 0.02). All drugs conditions, with the exception of PLACEBO, increased diastolic BP from pre- to post- drug. At the post-drug time point, this manifested in greater diastolic BP under TOB (MDiff: 6.33, s.e.: 1.61, *p* < 0.001) and CAN (MDiff: 6.22, s.e.: 1.61, *p* < 0.001) than CAN-TOB (MDiff: 1.26, s.e.: 1.61, *p* = 0.44). There was also a cannabis × tobacco interaction, which was explained/subsumed by the above three-way interaction (*F*_1,161_ = 5.70, *p* = 0.2). Finally, there was a cannabis × time interaction (*F*_1,161_ = 4.64, *p* = 0.03), which revealed a significant that active cannabis increased diastolic blood pressure, pre- to post-drug (MDiff: 4.51, s.e.: 1.13; *p <* 0.001), but not under placebo cannabis. There was also a main effect of time (*F*_1,161_ = 11.91, *p* = 0.001). There were no other main effects or interaction. For systolic blood pressure, a cannabis × tobacco interaction (*F*_1,161_ = 4.65, *p* = 0.03) emerged however pairwise comparisons revealed no significant differences between cannabis and placebo or between pre- and post-drug timepoints.

### Self-ratings

#### Stoned (Fig. 4*e*)

There was a cannabis × time interaction (*F*_1,161_ = 84.59, *p* < 0.001), which revealed a significant increase pre- to post-drug for active cannabis (MDiff: 4.95, s.e.: 0.31; *p* < 0.001) and for placebo cannabis to a lesser extent (MDiff: 0.91, s.e.: 0.31; *p* = 0.004). There was no difference between placebo and active cannabis pre-drug, however, there was a significant difference post- drug (MDiff: 4.02, s.e.: 0.31; *p* < 0.001). There was also a main effect of cannabis (*F*_1,161_ = 82.85, *p* < 0.001) and a main effect of time (*F*_1,161_ = 178.25, *p* < 0.001). There were no main effects or interactions with tobacco.

#### Dizzy (Fig. 4*f*)

There was a cannabis × time interaction (*F*_1,161_ = 17.07, *p* < 0.001), which revealed a significant increase pre- to post-drug for active cannabis (MDiff: 2.41, s.e.: 0.27; *p <* 0.001) and for placebo cannabis to a lesser extent (MDiff: 0.83, s.e.: 0.27; *p* = 0.002). Pre-drug, there was no difference between active and placebo cannabis (*p* = 0.817) however active cannabis increased ‘dizzy’ ratings post-drug (MDiff: 1.51, s.e.: 0.27, *p <* 0.001). There were significant main effects of cannabis (*F*_1,161_ = 17.46, *p <* 0.001), and time (*F*_1,23_ = 29.15, *p* < 0.001). No tobacco × time or cannabis × tobacco × time interactions emerged.

## Discussion

In the first study to investigate the acute interaction between cannabis and tobacco using a controlled randomised crossover design with an ecological method of drug administration, we found that cannabis impairs episodic memory. We found preliminary evidence to support our hypothesis that tobacco would offset the effects of cannabis on verbal recall. However, this finding emerged for delayed but not immediate recall, and was not supported by linear mixed model analysis, so should be treated with caution until replicated. When active tobacco is combined with active cannabis the impairment in delayed recall is slightly attenuated in comparison with cannabis alone. In regards to WM, we saw opposite independent effects whereby cannabis was detrimental to WM, and tobacco improved working memory performance. We also found that tobacco had no effect on cannabis-induced psychotic-like experiences. In regards to physiological effects, all drug conditions apart from the placebo increased diastolic BP post-drug. Diastolic BP was lower under mixed cannabis and tobacco than either cannabis alone or tobacco alone. The biological mechanisms of this effect are uncertain, but warrant further investigation, as mixed tobacco and cannabis is the primary route of self-administration. Both cannabis and tobacco had independent effects on HR, with cannabis producing greater increases in HR than tobacco. Tobacco did not influence ratings of ‘stoned’ or ‘dizzy’, which are classic cannabis-induced effects. Taken together, we found minimal evidence for interactive effects of cannabis and tobacco in a controlled 2 × 2 design with an ecological method of drug administration. However, our results tentatively suggest that the common practise of adding tobacco to cannabis in joints (Hindocha *et al.* 2016) may reduce cognitive impairment from cannabis, but does not influence users’ psychotic-like experiences or subjective experience of the drug.

Previous research has shown that cannabis acutely induces robust cognitive deficits in working and episodic memory (Curran *et al.* 2002; D'Souza *et al.* 2004; Morrison *et al.* 2009; Bossong *et al.* 2012). Tobacco has been shown to have the opposite effect on the same cognitive constructs but with much smaller effect sizes (Heishman *et al.* 2010) and both drugs act on receptors that densely populate the hippocampus. The *a priori* comparison on a prose recall task show, although there was no cannabis × tobacco interaction in the linear mixed model analysis, participants performed significantly better after cannabis and tobacco combined than cannabis alone for delayed recall (mean difference: 1.75 idea units) but not for immediate recall. These findings are similar to Englund *et al.* (2013) who found that THC-induced impairments in delayed but not immediate recall were attenuated by pre-treatment of CBD (Englund *et al.* 2013). Together, the prose recall and N-back results suggest that tobacco/nicotine increased attentional resources that may be involved in trying to recall information that had previously been encoding correctly. The delayed recall task is more difficult and requires greater attentional resources than the immediate recall task, and these results are in line with the general improvement effect found on the N-back. These results are also consistent with a recent study of chronic cannabis use, which found a cannabis × tobacco interaction for delayed recall. However, this effect was only evident among those who consistently smoked cigarettes (>100 per year) in comparison with those who sporadically smoked cigarettes (<100 per year) (Schuster *et al.* 2015). However, this study did not use a controlled design, used a relatively arbitrary cut-off for cigarettes and could not investigate adding tobacco to cannabis in the same product.

In regards to WM, we found the detrimental effect of cannabis (in comparison with placebo) on the N-back were load-dependent i.e. impairment increased with load, and was selective to manipulation (not maintenance). By contrast, facilitative effects of tobacco on correct responses and discriminability were load-independent, and did not influence manipulation or maintenance, perhaps suggesting that tobacco effects are purely on attention. This is consistent with previous function magnetic resonance imaging (fMRI) research showing nicotine altered activity in a neural network associated with task monitoring and attention (Kumari *et al.* 2003). Our results are consistent with a recent naturalistic study (Schuster *et al.* 2016), which used a 40-s WM task on mobile phones and found WM was impaired by cannabis, improved by tobacco, and when used simultaneously, participants showed no impairment. Moreover, both Schuster *et al.* (2016) and the present study did not find evidence for a cannabis × tobacco interaction for WM performance. In the present study *a priori* comparisons between cannabis + tobacco and cannabis alone were only approaching significance for *d*′. Our findings complement those of Schuster *et al.* (2016) and provide impetus for further investigation into the interactive effects of cannabis and tobacco on cognition. This study may also provide some mechanistic insights into memory and why both substances may be co-administered however, it would be essential to replicate this finding in another controlled study. One potential consequence of nicotinic attenuation of the effects of THC on memory may be that it feeds into continued drug taking as certain acute adverse effects are diminished. These results may have relevance to dual diagnosis populations, for whom rates of both cigarette and cannabis (and tobacco), use are high, presenting an important line of future research.

Tobacco had no effect on feeling ‘stoned’ or ‘dizzy’ despite this strongly-held belief that adding tobacco to cannabis increases positive subjective effects (Amos *et al.* 2004). Although tobacco potentially offset some of the impairing effect of cannabis on memory, this occurred in absence of any positive subjective effects. This is in contrast to previous human experimental research, which found that nicotine patch pre-treatment increased reports of feeling stimulated and an amphetamine-like feelings scale (Penetar *et al.* 2005). However, we found a cannabis × tobacco interaction on diastolic BP, and independent effects of cannabis and tobacco on heart rate, which suggest that combining the two, increases the cardiovascular risk of smoking cannabis (for diastolic BP, the combined was lower than cannabis alone and tobacco alone, however this does not negate the increase in diastolic BP). There is a clear public health implication here, suggesting that smoking cannabis with tobacco does not improve the subjective effects of cannabis, and makes it more harmful to one's physical health.

In relation to the PSI results, we found no modification of PSI scores by either tobacco alone or in combination with cannabis. This corresponds to research that finds nicotine also fails to attenuate ketamine-induced psychotic-like experiences and cognitive deficits (D'Souza *et al.* 2012). In recent epidemiological studies, tobacco and cannabis have been shown to independently predict the rate of psychotic-like experiences (Van Gastel *et al.* 2013; Gage *et al.* 2014). However, the relationship between cannabis, tobacco, and psychosis is complicated given tobacco and cannabis are so strongly correlated. These findings do not negate a possible long-term effect of tobacco on psychosis. However, they suggest that such an association is less biologically plausible than for cannabis, as evidenced by acute drug effects.

### Strengths and limitations

Strengths of this study include a large sample size (informed by an *a priori* power calculation), its double-blind, randomized, double-placebo-controlled, crossover design, and use of well-validated tasks. Furthermore, we selected participants with minimal dependence on tobacco (and cannabis), which suggests that nicotinic facilitation was not purely due to the reversal of withdrawal effects. We used the PSI, which has better test-retest reliability than other scales designed to tap psychotic like effects (De Simoni *et al.* 2013). Pharmacokinetics (PK)/pharmacodynamics(PD) were not measured so we are unable to comment on temporal changes that occur. For example, previous research has shown that nicotine increases the length of the cannabis effect in some participants (Penetar *et al.* 2005). Furthermore, nicotine effects reduce quickly after administration (Mendelson *et al.* 2003, 2005) in comparison with the cannabis effect and we were not able to conduct multiple dosing studies or ideally, an intravenous study (D'Souza *et al.* 2012) however the short-testing window was designed to capture nicotine's effects. Finally, given the novelty of the research, with multiple statistical comparisons study of cannabis and tobacco, we would suggest that these findings be treated with caution until replicated.

## Conclusions

In conclusion, this study found that cannabis impaired working and episodic memory. We found preliminary evidence that tobacco co-administration may offset the effects of cannabis on episodic memory. We characterised the acute subjective and cardiovascular effects of cannabis and tobacco administered together through a shared route of administration (i.e. joints) and found that these effects were similar to cannabis alone. There was no effect of tobacco on cannabis induced psychotomimetic effects.

## Supplementary material

For supplementary material accompanying this paper visit http://dx.doi.org/10.1017/S0033291717001222. click here to view supplementary material
