# Supplementary material for: Acute memory and psychotomimetic effects of cannabis and tobacco both ‘joint’ and individually: a placebo-controlled trial
Source: Psychol Med. 2017 May 31;47(15):2708–19. doi: 10.1017/S0033291717001222 (PMC5647681; doi:10.1017/S0033291717001222)
Supplement: Supplementary file 1 [file S0033291717001222sup001.docx]

**SUPPLEMENTARY MATERIALS**

**Acute memory and psychotomimetic effects of cannabis and tobacco both ‘joint’ and individually: a placebo-controlled trial**

Chandni Hindocha^1^, Tom P Freeman^1^, Richard Xia^2^, Natacha D C Shaban^1^, H. Valerie Curran^1^

Methods

## Participant Recruitment

Participants were recruited from the community through posters around universities of London and on online notice boards.

## Smoking Procedure

The smoking procedure was standardised to control for dose titration and maximise absorption of THC (1). Participants were asked to inhale for 4 seconds, hold their breath for 8 seconds, and then exhale and break for 30 seconds. This sequence was repeated until the joint were smoked up to a designated line (Fig. 1). This protocol was timed and enforced by the experimenter.


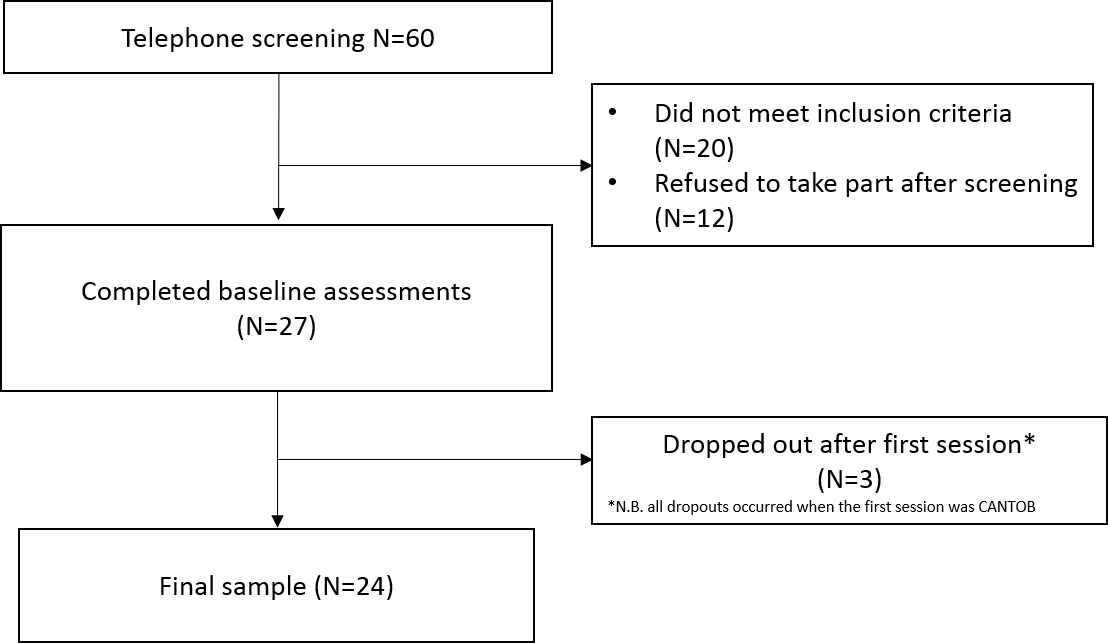


*Supplementary Figure S1: Flowchart of participant recruitment. Participants were telephone screened, if inclusion was met, they completed the baseline and first session. 3 participants did not complete the first session and were replaced to meet the final sample of 24 as deemed appropriate from our a priori power calculation.*

*
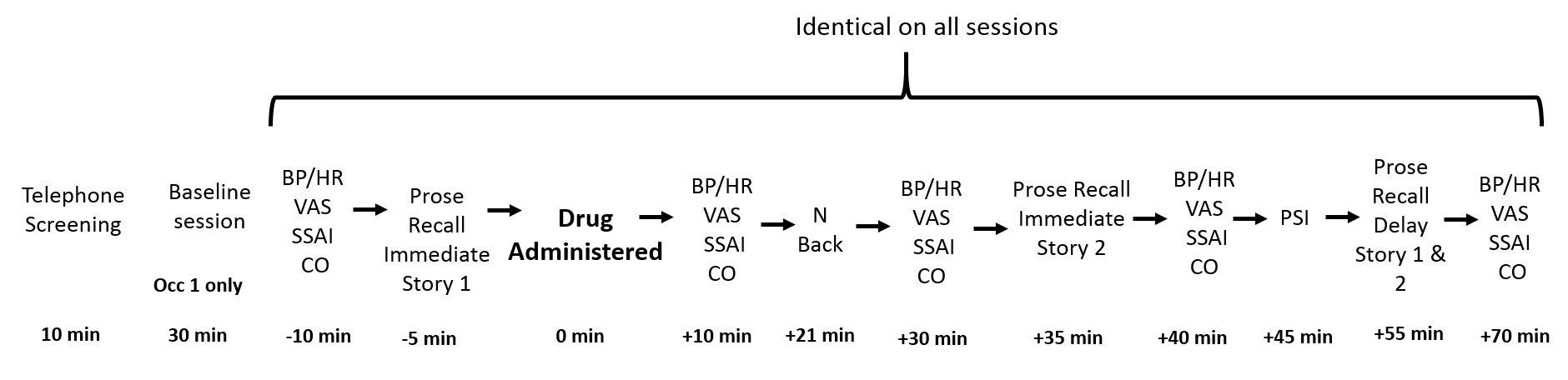
*

*Supplementary Figure S2: Flowchart of Assessments. Other tasks that are not reported here were undertaken at the intervening time points. Drug administration timings can be seen in Suppl. Table S2. Post-drug timings are from the beginning of smoking onset. The following abbreviations are used - BP: Blood pressure, HR: Heart Rate, VAS: Visual Analogue Scales, SSAI: Short State Anxiety Inventory, CO: Carbon Monoxide, PSI: Psychotomimetic States Inventory.*

Results

## Drug History (Supplementary Table S1)

*Supplementary Table S1: Drug use history (the number of participants varied if they had ever used that drug). Use of other drugs was minimal except for alcohol.*

|  | Alcohol (N=24) | Tobacco (N=24) | Cannabis alone (N=23) | Cannabis+  Tobacco (N=24) | Cocaine  (N=17) | MDMA (N=18) |
| --- | --- | --- | --- | --- | --- | --- |
| Last used (days) | 3.46 ± 3.21 | 96.125 ± 313.26 | 466.86 ± 866.37 | 7.92 ± 9.64 | 198.46 ± 359.89 | 295.44 ± 851.03 |
| Age of first use (years) | 13.12 ± 2.40 | 15.71 ± 1.94 | 16.32 ± 5.41 | 16.16 ± 3.94 | 19.80 ± 2.67 | 19.05 ± 2.98 |
| Years used (years) | 9.04 ± 4.57 | 6.76 ± 4.58 | 3.31 ± 4.16 | 6.79 ± 3.94 | 4.34 ± 3.17 | 5.50 ± 5.10 |
| Days per month | 8.8 ± 5.48 | 11.04 ± 12.68 | 0.82 ± 2.09 | 7.75 ± 4.43 | 0.41 ± 0.66 | 0.13 ± 0.34 |
| Amount consumed* | 5.88 ± 2.68 | 2.29 ± 2.74 | - | 36.58 ± 34.47 | 331.33 ± 164.78 | 298.75 ± 240.82 |
| Lifetime exposures (days) | 821.04 ± 556.66 | 2834 ± 7202 | 49.18 ± 97.60 | 626.83 ± 935.51 | 34.65 ± 61.57 | 42.40 ± 63.92 |
| Exposures in the last 90 days (days) | 24.29 ± 17.93 | 29.75 ± 33.56 | 3.55 ± 6.39 | 19.58 ± 11.27 | N/A | N/A |

*_Notes: *For Amount consumed the units vary – Alcohol: units per session; Tobacco: cigarettes per day; Cannabis+Tobacco: Time to smoke an eighth (3.5g) (days); Cocaine: mg; MDMA: mg. Cannabis alone is missing as participants would not smoke a standard 3.5g in this manner._*

## Time to smoke study joints (Supplementary Table S2)

There were no significant differences between drug conditions on time (minutes) to smoke the joint on each day or number of puffs (see Supplementary Table S2).

*Supplementary Table S2: Mean time (mins) to smoke the joint and number of puffs for each drug condition.*

|  | PLACEBO | TOB | CAN | CAN-TOB | Test Statistic |
| --- | --- | --- | --- | --- | --- |
| Time to smoke joint (mins) | 7.17 ± 1.76 | 7.95 ± 2.82 | 6.78 ± 1.17 | 7.33 ± 1.40 | *F*_3,56_=2.80, *p=*0.06 |
| Puffs taken | 6.96 ± 1.12 | 7.41 ± 1.41 | 7.08 ± 1.47 | 7.27 ± 1.30 | F_3,63_=0.80 *p=*0.50 |

*Supplementary Table S3: Means (SEM) for Number of correct responses, Reaction Time (RT), D prime (d’) and Criterion (C) for each drug condition.*

|  | PLACEBO | | | TOB | | | CAN | | | | CAN-TOB | | | |
| --- | --- | --- | --- | --- | --- | --- | --- | --- | --- | --- | --- | --- | --- | --- |
|  | 0 back | 1 back | 2 back | 0 back | 1 back | 2 back | 0 back | 1 back | 2 back | 0 back | | 1 back | 2 back |  |
| Number Correct | 47.92 (0.22) | 43.63 (0.59) | 39.83 (1.22) | 47.54 (0.63) | 44.75 (0.52) | 42 (0.98) | 45.83 (0.87) | 42.08 (0.95) | 36.21 (1.62) | 47.83 (0.28) | | 43 (1.02) | 37.54 (1.57) |  |
| RT | 417.58 (13.13) | 532.96 (26.42) | 613.02 (28.52) | 457.35 (34.34) | 535.08 (29.60) | 610.16 (34.57) | 445.94 (27.81) | 577.50 (27.94) | 701.03 (44.35) | 429.99 (28.04) | | 542.61 (32.2) | 675.12 (43.18) |  |
| d’ | 3.19 (0.07) | 2.99 (0.13) | 2.27 (0.20) | 3.15 (0.12) | 3.22 (0.14) | 2.64  (0.17) | 2.66 (0.22) | 2.67 (0.19) | 1.91 (0.23) | 3.14 (0.9) | | 3.07 (0.17) | 1.99 (0.20) |  |
| C | 0.51 (0.03) | -0.08 (0.03) | -0.04 (0.05) | 0.52 (0.04) | -0.01 (0.04) | -0.11 (0.05) | 0.52 (0.04) | -0.01 (0.04) | -0.02 (0.05) | 0.44 (0.04) | | -0.06 (0.04) | -0.08 (0.04) |  |

**References**

1. Ramaekers JG, Kauert G, van Ruitenbeek P, Theunissen EL, Schneider E, Moeller MR (2006): High-potency marijuana impairs executive function and inhibitory motor control. *Neuropsychopharmacology*. 31:2296-2303.
